# Getting the Fluid Balance Right in Malaria

**DOI:** 10.1371/journal.pmed.0010024

**Published:** 2004-10-19

Acidosis is a major cause of death in patients with malaria, although what causes acidosis is still unclear. One possibility is that hypovolemia contributes to the problem, and that rehydration therapy could be of benefit. Now, Sanjeev Krishna and colleagues have shown that in children with severe malaria dehydration is not severe and is not correlated with other measures of disease severity. “The optimum resuscitation approach in severe childhood malaria remains to be defined,” says Nick White (Mahidol University, Thailand), the academic editor of the paper. “The relative advantages of blood, colloids, and crystalloids need to be characterized.”[Fig pmed-0010024-g001]

**Figure pmed-0010024-g001:**
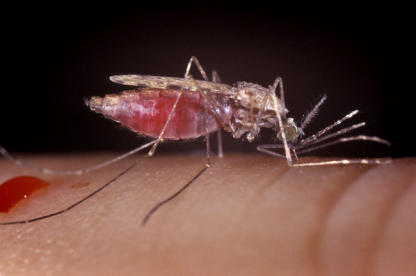
Anopheles gambiae, the principal vector of malaria (Photo: Jim Gathany)

Every year around 200 million people worldwide contract malaria, of whom over a million die. The vast majority of those who die are children under five years, mostly in Africa, since young children have had little chance to acquire any immunity. Fluid resuscitation is generally considered to be a cornerstone of treatment—but how much fluid should be given? Some researchers believe that surrogate signs of fluid depletion—such as tachycardia, reduced capillary refill time, and reduced urine excretion—suggest that there is substantial volume depletion. The reason that the amount of fluid given matters so much is that giving too much, especially of hypotonic solutions, can lead to electrolyte imbalance, especially hyponatremia and hypokalemia.

Research efforts have been hampered by not having an easy way to assess in patients the fluid depletion in different compartments of the body, i.e., total body water and extracellular and intracellular water volume. Krishna and colleagues used heavy-water distribution to calculate the total body water and bromide distribution to determine the extracellular volume in 19 children with moderately severe malaria and 16 with severe malaria in Gabon. By subtracting extracellular volume from total body water, they were able to calculate intracellular volume for each child. They also used a less invasive and more rapid method of determining water volumes based on using bioelectrical impedance to calculate the volume.

None of the children were severely dehydrated (defined as more than 100 ml/kg depletion), and only three of the children with severe anemia had fluid depletion, which was moderate (60–90 ml/kg depletion). “This challenges the view that dehydration is a major contributor to the pathology of this frequently lethal disease,” says White.

So based on these data, obtained from a carefully studied, albeit small group of children, what should people who treat children with malaria do? The authors' first recommendation is that clinicians should think again about how vigorously they rehydrate children, and if they have access to ways of assessing fluid volume more precisely, they should do so (not a trivial undertaking in many hospitals where these children are treated). And certainly the methods used by Krishna and colleagues should undergo wider testing in larger groups of children to confirm their usefulness. Until the worldwide efforts to prevent malaria come to fruition, refining the management of infected children will remain a cornerstone of the efforts against this devastating disease.

